# Does regular Khat (*Catha edulis*) chewing in adults increase the risk of hypertension compared to non-use? A systematic review and meta-analysis

**DOI:** 10.1186/s12872-025-05160-2

**Published:** 2025-10-21

**Authors:** Abdulkerim Hassen Moloro, Abubeker Alebachew Seid, Molla Getie Mehari, Abdu Hailu Shibeshi, Bizunesh Fantahun Kase

**Affiliations:** 1 Department of Nursing, College of Medicine and Health Sciences, Samara University, Samara, Ethiopia; 2 Department of Medical Laboratory Science, College of Medicine and Health Sciences, Injibara University, Injibara, Ethiopia; 3 Department of Statistics, College of Natural and Computational Sciences, Samara University, Samara, Ethiopia; 4 Department of Public Health, College of Medicine and Health Sciences, Samara University, Samara, Ethiopia

**Keywords:** Hypertension, Khat, *Catha Edulis*, Khat chewing

## Abstract

**Background:**

Global khat (*Catha edulis*) consumption currently affects approximately 20 million individuals, despite originating primarily in East Africa and the Arabian Peninsula. While traditionally viewed as a regional practice with limited relevance to Western societies, modern distribution networks and diaspora communities have facilitated its international spread, transforming khat into a global substance of concern. This study builds upon existing evidence by synthesizing data from fifteen studies which is significantly more than previous reviews to firmly establish khat (*Catha edulis*) use as a risk factor for elevated blood pressure and tachycardia, both of which are key precursors to hypertension.

**Objective:**

This systematic review and meta-analysis aimed to aimed to systematically evaluate, and synthesize the available evidence on the epidemiological association between khat (*Catha edulis*) chewing as an exposure and hypertension as an outcome, 2024.

**Methods:**

The comprehensive search of this systematic review and meta-analysis included cross-sectional and case-control studies published in English from inception up to December 30, 2024 on association between khat (*Catha edulis*) and hypertension. Excluded were conference proceedings, qualitative research, commentaries, editorial letters, case reports, case series, and monthly and annual police reports. The search covered full-text publications written in English and databases such as PubMed/MEDLINE, African Journals Online (AJOL), and Google Scholar. A checklist from the Joanna Briggs Institute (JBI) was used to evaluate the quality of the studies. Two independent reviewers performed data extraction, critical appraisal, and article screening. Assessment of the certainty evidences was done by applying the GRADE method. The statistical analysis was done using the software packages STATA-17 and RevMan 5.4. The Cochrane Q statistic with I^2^ was used to assess between study heterogeneity. A weighted inverse variance random effects model was used to calculate the pooled odds ratio with 95% confidence interval.

**Results:**

Fifteen studies were included in this systematic review and meta-analysis. From meta-analysis, a total of 12,409 participants were involved. Of the 3986 Khat chewers, 1278 were found to have hypertension. On contrary, out of the 8423 of non-chewers, 1341 were found to have hypertension. Pooled analysis showed that khat chewer were 2.4 times more likely to have hypertension as compared to non-khat chewer (OR 2.4, 95%CI 1.48–3.88) at *p* = 0.0004, I^**2**^ = 93% (95% CI: 88–96%). The findings of the Egger’s test (*P* = 0.655) and funnel plot revealed that no evidence of publication bias.

**Conclusion:**

This systematic review suggests khat chewing as a significant modifiable risk factor for hypertension, urging targeted public health interventions, policy regulation, and integration of cessation counseling into healthcare programs. Future research should employ rigorous designs, standardized measurements, and broader literature inclusion to strengthen causal evidence, while collaboration with endemic regions and objective biomarker use could enhance data reliability and generalizability. Addressing khat use may substantially reduce hypertension-related cardiovascular burdens in affected populations.

**Trial registration:**

This systematic review and meta-analysis was registered in PROSPERO with the registration ID and link as follows: CRD42024555322: Available from: https://www.crd.york.ac.uk/prospero/display_record.php.

**Supplementary Information:**

The online version contains supplementary material available at 10.1186/s12872-025-05160-2.

## Introduction

Hypertension is the result of persistently high blood pressure in the arteries. It is specifically described as having two or more readings of ≥ 140 mm Hg for the systolic blood pressure or ≥ 90 mm Hg for the diastolic blood pressure [1, 2]. In the world, it is the primary preventable cause of heart disease, disability (including damage to the heart, kidneys, eyes, brain, and large and peripheral blood arteries resulting from stroke) [3], and mortality [4].

Worldwide, 1.28 billion persons between the ages of 30 and 79 have hypertension, with over two thirds of those affected living in low- and middle-income nations [5–7]. It is predicted that 1.5 billion individuals globally will suffer from hypertension by 2025. Currently, 74.7 million people in Sub-Saharan Africa suffer from hypertension; by 2025, that figure is predicted to increase to 125.5 million [8]. Furthermore, projections show that 216.8 million Africans will suffer from hypertension by 2030 [9–11]. The number of DALYs (disability-adjusted life-years) lost due to HTN increased from 95.9 million to 143.0 million [12].

Khat (*Catha edulis*) is a plant that grows naturally in countries bordering the Red Sea, as well as along the east coast of Africa and the Arabian Peninsula. An estimated 20 million individuals worldwide currently use khat [13]. While initially considered a regional concern, modern distribution networks and diaspora communities have facilitated its global spread [14]. Prevalence rates remain particularly high in traditional use regions, reaching 15% in Ethiopia and up to 90% in Yemen [15].

Epidemiological evidence indicates that khat consumption is linked to higher rates of cardiovascular issues [16–18], including stroke [19–21], hypertension [22, 23], myocardial infarction and cardiomyopathy [24, 25]. Fresh khat leaves contain more than 40 bioactive compounds, with the amphetamine-like alkaloids cathine and cathinone primarily mediating its central nervous system and cardiovascular effects [26]. A study reported that 100 g of Khat leaves contain approximately 114 mg of cathinone, 83 mg of cathine, and 44 mg of nor-ephedrine [22, 27]. These compounds exert significant cardiovascular impacts, including increased heart rate, elevated blood pressure, and vasomotor changes in coronary vessels [28]. Experimental studies demonstrate that cathinone administration produces marked increases in both blood pressure and heart rate [17], while chronic khat consumption has been consistently associated with sustained elevations in these parameters. These hemodynamic changes appear dose-dependent, correlating with plasma cathinone concentrations [29].

The increased heart rate and blood pressure observed in individuals who chew Khat are primarily due to the enhanced sympathetic activity of cathinone. Cathinone, the main active ingredient in Khat, is responsible for elevating blood pressure. Regular Khat consumption leads to persistent cathinone levels in the bloodstream, resulting in prolonged vasoconstriction and chronically elevated blood pressure [30–32]. Frequent Khat chewers experience systolic blood pressure levels that are fourteen times higher than those of less frequent chewers [33]. Additionally, the duration of Khat chewing and the number of hours spent per session appear to be directly associated with hypertension. Individuals who spend more than 6 h in a Khat chewing session are almost 9 times more likely to have elevated diastolic blood pressure compared to those who spend less time chewing [34, 35].

This study incorporates fifteen studies, significantly expanding upon previous reviews that examined khat as a risk factor for hypertension (3 studies) [36] and cardiovascular disorders (6 studies) [37]. This larger evidence-based studies than previous studies enabled us to establish khat use as a significant risk factor for both elevated blood pressure and tachycardia - well-documented precursors to hypertension. These findings resolve a key limitation identified in Kalkidan et al.‘s review [36], which had concluded there was insufficient evidence to link khat consumption with hypertension. Therefore, this systematic review and meta-analysis aims to provide the pooled association of hypertension and khat chewing among khat chewer individuals. The findings may help health professionals, health planners and policymakers as well as the community themselves to prevent risk factors for hypertension alarming rise.

### Objectives and review questions

The objective of this systematic review and meta-analysis aimed to systematically evaluate, and synthesize the available evidence on the epidemiological association between khat chewing as an exposure and hypertension as an outcome. The following review questions provide a framework for this systematic review and meta-analysis based on the PICO format: 1) Does regular khat chewing in adult populations compared to non-use increase the risk of developing hypertension based on available epidemiological evidence?

## Methods

### Study report and registration

This systematic review and meta-analysis adhered to the guidelines outlined by the Preferred Reporting Items for Systematic Reviews and Meta-Analyses (PRISMA) statement [38]. The study results were reported following the PRISMA-2020 standard [38]. Additionally, the study protocol was registered with the International Prospective Register of Systematic Reviews (PROSPERO) under the registration identification number CRD42024555322.

## PICO search guide

### Population

Adult khat chewer.

### Intervention/Exposure

Khat chewing.

### Comparison

Non-use of khat.

### Outcome/Condition

Risk of developing hypertension.

### Search strategy and sources of information

Two independent authors (BFK and AHM) designed and executed the search strategy. Published research on the association between hypertension and khat chewing among khat chewer was used in the review. A systematic search was conducted for studies published from inception up to December 30, 2024 across multiple electronic databases. The search yielded the following results: Google Scholar (33 studies), African Journals Online (AJOL) (38 studies), PubMed/MEDLINE (36 studies), and Google/citation searches (68 studies). All relevant records were included for further screening. As a result of the comprehensive search and further screening, only studies published between 2002 and 2023 were included in the review.

For the PubMed advanced search, a combination of keywords, free-text terms, and Medical Subject Headings (MeSH) was used, linked with Boolean operators (see Table 1). The same search strategy, incorporating these terms, was applied across PubMed, Google Scholar, and AJOL: *[“Khat” OR “qat” OR “Khat chewing”*,* OR “qat chewing”*,* OR “Khat chewers” OR “qat chewers” OR “chata edulis”*,* OR “effect of khat” OR “effect of Qat” OR “effect of chata edulis” OR “hypertension”*,* OR “blood pressure”*,* OR “Man”*,* OR “Adult”*,* OR “Person”]/[“Khat” AND“qat”AND “Khat chewing”*,* AND “qat chewing”*,* AND “Khat chewers” AND “qat chewers” AND “chata edulis”*,* AND “effect of khat” AND “effect of Qat” AND “effect of chata edulis” AND “hypertension”*,* AND “blood pressure”*,* AND “Man”*,* AND “Adult”*,* AND “Person”]*.

To ensure a thorough and comprehensive study, we consulted an experienced librarian for guidance. We employed a snowballing technique, examining references from identified publications and mining citations from previous narrative and systematic reviews to uncover additional relevant studies. Additionally, to ensure a thorough search and minimize publication bias, efforts were made to identify unpublished or non-commercially published research, including theses, dissertations, government reports, and clinical trial registries. Furthermore, we sought recommendations from specialists, researchers, and relevant organizations to identify another pertinent research that had already been conducted. However, no additional relevant studies meeting the inclusion criteria were found or retrieved from these sources.

The search results from Google Scholar, African Journals Online (AJOL), and PubMed/MEDLINE were imported into the reference management software (Endnote™) where duplicate entries were removed to ensure efficiency and avoid redundancy. This multi-faceted approach helped us gather a robust and well-rounded collection of studies for our research.

**Table 1:** PubMed search strategy for systematic review and meta-analysis on the pooled association between hypertension and khat chewer, 2024

Search number	Search Details	Results
1	“hypertension“[MeSH Terms]	***325***,***011***
2	“Blood pressure high“[Title/Abstract] OR “blood pressures high“[Title/Abstract] OR “high blood pressure“[Title/Abstract] OR “high blood pressures“[Title/Abstract]	***21***,***422***
3	“Catha“[MeSH Terms]	***663***
4	“Catha edulis“[Title/Abstract] OR ((“Catha“[MeSH Terms] OR “Catha“[All Fields]) AND “eduli“[Title/Abstract]) OR “edulis Catha“[Title/Abstract] OR “Khat“[Title/Abstract] OR “Mairungi“[Title/Abstract] OR “Miraa“[Title/Abstract] OR “qat plant“[Title/Abstract] OR “plant qat“[Title/Abstract] OR ((“plant s“[All Fields] OR “planted“[All Fields] OR “planting“[All Fields] OR “plantings“[All Fields] OR “Plants“[MeSH Terms] OR “Plants“[All Fields] OR “Plant“[All Fields]) AND “Qat“[Title/Abstract]) OR (“Qat“[All Fields] AND “Plants“[Title/Abstract])	***1***,***328***
5	“hypertension“[MeSH Terms] OR “blood pressure high“[Title/Abstract] OR “blood pressures high“[Title/Abstract] OR “high blood pressure“[Title/Abstract] OR “high blood pressures“[Title/Abstract]	***334***,***105***
6	“Catha“[MeSH Terms] OR (“Catha edulis“[Title/Abstract] OR ((“Catha“[MeSH Terms] OR “Catha“[All Fields]) AND “eduli“[Title/Abstract]) OR “edulis Catha“[Title/Abstract] OR “Khat“[Title/Abstract] OR “Mairungi“[Title/Abstract] OR “Miraa“[Title/Abstract] OR “qat plant“[Title/Abstract] OR “plant qat“[Title/Abstract] OR ((“plant s“[All Fields] OR “planted“[All Fields] OR “planting“[All Fields] OR “plantings“[All Fields] OR “Plants“[MeSH Terms] OR “Plants“[All Fields] OR “Plant“[All Fields]) AND “Qat“[Title/Abstract]) OR (“Qat“[All Fields] AND “Plants“[Title/Abstract]))	***1***,***376***
7	(“hypertension“[MeSH Terms] OR (“blood pressure high“[Title/Abstract] OR “blood pressures high“[Title/Abstract] OR “high blood pressure“[Title/Abstract] OR “high blood pressures“[Title/Abstract])) AND (“Catha“[MeSH Terms] OR (“Catha edulis“[Title/Abstract] OR ((“Catha“[MeSH Terms] OR “Catha“[All Fields]) AND “edulis“[Title/Abstract]) OR “edulis Catha“[Title/Abstract] OR “Khat“[Title/Abstract] OR “Mairungi“[Title/Abstract] OR “Miraa“[Title/Abstract] OR “qat plant“[Title/Abstract] OR “plant qat“[Title/Abstract] OR ((“plant s“[All Fields] OR “planted“[All Fields] OR “planting“[All Fields] OR “plantings“[All Fields] OR “Plants“[MeSH Terms] OR “Plants“[All Fields] OR “Plant“[All Fields]) AND “Qat“[Title/Abstract]) OR (“Qat“[All Fields] AND “Plants“[Title/Abstract])))	***36***

### Selection of studies

The articles retrieved from electronic database searches were imported into EndNote X7 reference management software, which automatically eliminated duplicate records. Two independent reviewers (MGM and BFK) then screened the remaining titles and abstracts based on predefined inclusion criteria. The screening process followed a structured methodology: first, article titles were assessed for relevance; next, eligibility was determined through an abstract review using predetermined inclusion and exclusion criteria; finally, the selected abstracts underwent a full-text screening. Data extraction and screening were completed using Microsoft Excel™ to ensure an organized and systematic approach.

To evaluate the consistency of assessments among multiple authors, inter-rater agreement was measured in accordance with the Cochrane Handbook for Systematic Reviews [39]. A kappa value of 0.75 or higher was deemed indicative of excellent agreement. Two independent authors (AAS and AHM) conducted a full-text review of the screened articles, applying predefined inclusion criteria to assess their relevance for the review. In cases where additional information was needed to clarify eligibility, the remaining authors were consulted. Any disagreements regarding study inclusion were resolved through discussion. Additionally, detailed records of exclusion reasons were maintained at each stage of the screening process.

### Eligibility criteria

This systematic review examined the association between regular khat chewing and hypertension in adult populations by comparing khat chewer (regular or occasional) with non-users. Khat chewer defined as an individual who ever had chewed khat in the last month before the study and currently actively engaged in khat chewing [40]. The review included studies involving both male and female adults aged 18–65 years, specifically those who had chewed khat for at least four years, as well as non-chewers with no personal or family history of hypertension or prior khat use. The review also included only those studies in which participants refrained from consuming coffee, alcohol, or tobacco for at least four hours before data collection.

The primary outcome was an increase in heart rate or hypertension (defined as blood pressure ≥ 140/90 mmHg), resulting from cathinone-induced sympathetic activation. The review incorporated cross-sectional and case-control studies, including peer-reviewed journal articles and national surveys from various countries, regardless of study location. Although limited to English-language publications, the search encompassed all relevant studies from the inception of the research topic up to the present to ensure comprehensive coverage of available epidemiological evidence.

This review did not included studies that do not provide information on the association between hypertension and khat chewing, or for which it is not possible to obtain the necessary information even after contacting the authors. Studies not reporting hypertension or related measurable outcomes, studies without a comparator group or unclear comparison, and studies with non-quantified khat use without clear exposure definition were excluded. Conference proceedings, qualitative studies, commentary, editorial letters, case reports, case series, monthly and annual police reports, and articles published in languages other than English were excluded. Furthermore, studies involving pregnant women, individuals with mental health disorders, or chronic conditions such as renal disease and diabetes were excluded to reduce potential confounding factors.

### Data extraction and management

Once every eligible article had been found, the pertinent data was extracted onto a Microsoft Excel spreadsheet by two independent reviewers (AHM&BFK). The Joanna Briggs Institute (JBI) data extraction form for systematic reviews and research syntheses served as the model for the development of a data extraction format [41, 42]. The data extraction tool contained the following information for each included article: the last name of the first author, the year the study was published, country of the study conducted in, the study design, sample size and sampling characteristics, exposure (potential risk factor), criteria for blood pressure measurement and hypertension diagnosis, data collection methods, and number of khat chewer and non-khat chewer with event and without event as well as their total.

The inter-rater reliability between the data extractors was assessed using Cohen’s kappa coefficient, calculated based on a 30% random sample of the included studies [43]. The strength of agreement was categorized as follows: low (κ = 0.20), fair (κ = 0.21–0.40), moderate (κ = 0.41–0.60), good (κ = 0.61–0.80), or almost perfect (κ = 0.81–1.00). A kappa value of ≥ 0.5 was considered acceptable, indicating sufficient agreement. Any discrepancies between the two extractors were resolved through discussion and re-evaluation of the original articles, with unresolved disagreements adjudicated by a third reviewer (AAS).

### Quality assessment

The listed studies were assessed independently by two reviewers (AHS&MGM). The JBI checklists for analytical cross sectional study and case control study [44] were used to assess the articles’ quality. The tool for analytical cross sectional study has eight parameters: (1) a suitable sampling frame; (2) a description of the subject and setting of the study; (3) a description of the measurement of exposure in a valid and reliable way; (4) a description of the standard criteria used for measurement of the condition; (5) a description of the identification of the confounding factors; (6) strategies to deal with confounding factors; (7) valid measurement for each outcomes; and (8) the application of suitable statistical analysis.

The tool for case control study nine parameters: (1) a description of comparable groups other than the presence of disease in cases or the absence of disease in controls suitable sampling frame; (2) a comparable cases and controls or matched appropriately; (3) a same criteria used for identification of cases and controls; (4) a description of the measurement of exposure in a valid and reliable way; (5) a description of the exposure measurement in the same way for cases and controls;(6) a description of the identification of the confounding factors; (7) strategies to deal with confounding factors; (8) valid measurement for each outcomes; and 9) the application of suitable statistical analysis.

Alternatives such as yes, no, not applicable, and unclear are available in the tools. Responses that were yes received a score of one, while those that were unclear, not applicable, or no received a zero. A study was ultimately graded as low, medium and high quality if it scored less than 50%, between 50% and 70% and above 70% respectively [45, 46] and included in the final analysis. Throughout the critical evaluation, one author (BFK) settled the arguments between the two authors.

### Statistical analysis

The data were displayed using tables and graphs according to the findings of the selected study’s conclusions. Publication bias and sensitivity analysis assessed using STATA 17 version software. The random effect model was used to show the pooled association between hypertension and khat chewing among khat chewer when heterogeneity seen among studies and fixed effect model used when no heterogeneity seen [47, 48]. The pooled analysis was done using RevMan 5.4 version software. The number of khat chewer and non-khat chewer (case with event, case total, control with event, control total) were entered to calculate odd ratio (OR). Case were individuals who chewed khat and had hypertension; controls were individuals who never chewed khat.

Using the I^2^ statistic and a chi-squared test in accordance with Cochran’s Q statistic with a 5% significance level, heterogeneity was measured based on statistical findings, outcome presentations, and methodological [49]. I^2^ values of 0%, 25%, 50%, and 75% were considered indicative of no, low, moderate, and high heterogeneity, respectively [50]. Due to the observed heterogeneity between the studies, a weighted inverse variance random-effects model was used to calculate the pooled odds ratio with a 95% confidence interval. Furthermore, subgroup and sensitivity analysis were performed to investigate and minimize sources of heterogeneity [51].

#### Publication bias

For the purpose of examining the possibility of publication bias and small-study effects, funnel plots and Egger’s test [52] were utilized. Publication bias was identified when the p-value was statistically significant (p value < 0.05). To investigate publication bias, at least 10 studies are required [53]. As a result, the absence of publication bias was demonstrated by Egger’s linear regression test (*P* = 0.655) and funnel plot revealed symmetrical distribution.

#### Certainty assessment in cumulative evidence

The meta-analysis articles selected for this study were evaluated based on the quality of evidence, as determined by the five key domains of the GRADE (Grading of Recommendations, Assessment, Development, and Evaluation) framework. These domains include risk of bias, inconsistency in results, indirectness of evidence, imprecision of findings, and publication bias [54–56]. The overall quality of evidence for each study was then classified into one of four levels: high, moderate, low, or very low. To ensure objectivity, the GRADE evaluation of the articles was conducted independently by two authors (AHM and AHS). They independently assessed and verified the final ratings of the included studies, with any discrepancies resolved through discussion to reach a consensus.

## Results

### Study selection and identification

**Fig. 1:**
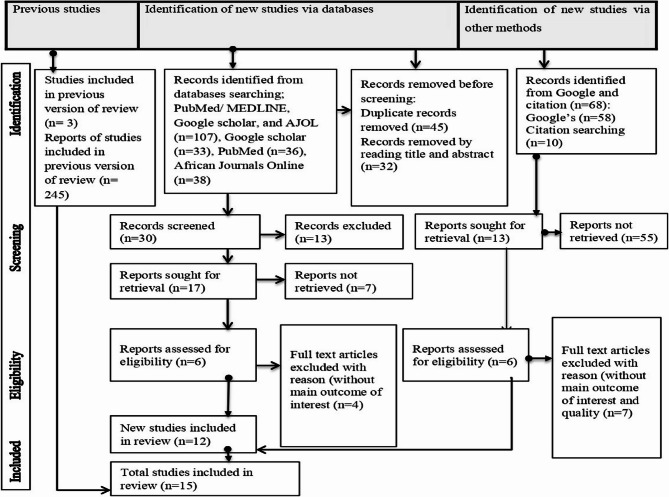
PRISMA (Preferred Reporting Items for Systematic Reviews and Meta-Analyses) flowchart

An electronic search using MEDLINE/PubMed, Google Scholar, and African Journal Online showed up 107 articles in the first search. Forty-five of which were eliminated due to duplication. After reading their titles and abstracts, 32 studies were removed from this review. A manual search of reference lists and Google yielded 68 articles. Out of 68 studies, 55 were excluded due to various reasons such as lack of full text and quality of methodology. Eventually, 15 publications [57–71] that satisfied all inclusion requirements were incorporated to the review and meta-analyses. The PRISMA flow diagram (See Fig. 1) includes information about the screening procedure.

### Assessment of methodological quality for included studies

There were 15 studies in this review with methodological quality ranging from moderate to high. According to the JBI critical appraisal checklist [72], which was used for studies that reported analytical cross sectional study, ten studies [57, 68, 69, 73–79] scored 8 points, three studies [58, 80, 81] scored 6 points, and the remaining 1 studies [34, 71, 82] scored 7 points. Additionally, one case control study [83] included in this review and according to the JBI critical appraisal checklist [44], which used for studies that reported case control study and it scored 9 points (See Table 2).

**Table 2:** Methodological quality of included for the association between Khat chewing and hypertension, 2024

Studies	Q1	Q2	Q3	Q4	Q5	Q6	Q7	Q8	Q9	Q10	Quality score
Getahun et al. [57]	✓	✓	✓	✓	✓	✓	✓	✓			**8**
Mossie et al. [58]	✓	✓	✓	✓	✓	✓	✓	X			**6**
Ibrahim et al. [80]	✓	✓	✓	✓	✓	X	✓	✓			**6**
Ayehu et al. [84]	✓	✓	✓	✓	✓	X	✓	X			**6**
Chukaeta et al. [73]	✓	✓	✓	✓	✓	✓	✓	✓			**8**
Geta et a. [74]	✓	✓	✓	✓	✓	✓	✓	✓			**8**
Gebremedhin et al. [83]	X	✓	✓	✓	✓	✓	✓	✓	✓	$\surd$	**9**
Badego et al. [75]	✓	✓	✓	✓	✓	✓	✓	✓			**8**
Yimam et al. [76]	✓	✓	✓	✓	✓	✓	✓	✓			**8**
Abdissa and Kene et al. [77]	✓	✓	✓	✓	✓	✓	✓	✓			**8**
Wachamo et al. T [78]	✓	✓	✓	✓	✓	✓	✓	✓			**8**
Sisay et al. [68]	✓	✓	✓	✓	✓	✓	✓	✓			**8**
Seifu W, et al. [69]	✓	✓	✓	✓	✓	✓	✓	✓			**8**
Motuma et al. [79]	✓	✓	✓	✓	✓	✓	✓	✓			**8**
Hassen and Mamo et al. [71]	✓	✓	U	✓	✓	✓	✓	✓			**7**

*Hint*: *✓ = Yes*,* X = No*,* U = Unclear*

*⎫ Fourteen studies are cross sectional studies and evaluated out of 8 using JBI critical appraisal for analytical cross-sectional studies and one study is case-control study and evaluated out of 10 using JBI critical appraisal for case-control study*

### Characteristics of included studies

This systematic review and meta-analysis included 15 original publications that were conducted in two nations. Among these, fourteen (93.33%) were conducted in Ethiopia [34, 57, 58, 68, 69, 71, 73–79, 81–83] and one study from Saudi Arabia [80]. Among the included studies [71, 73–79, 81–83], 10 (66.7%) studies published in 2019 and above. Regarding study design, fourteen (93.33%) studies were cross-sectional studies and one study [83] was unmatched case control. All included participants were adults aged 16 and above regardless of sex. All included studies used blood pressure measurement ≥ 140/90 mmHg and above. A total of 12,409 participants were included in this systematic review and meta-analysis. Out of 15 included studies, thirteen studies measured blood pressure at the time of the data collection using standardized blood pressure measurement and two studies [69, 75] used history of the hypertension diagnosis and antihypertensive drug taking at the time of data collection (See Table 3).

**Table 3:** Characteristics of included studies for the association between Khat chewing and hypertension, 2024

Author and year of publication	Country	Sample characteristics	Study design	Exposure (potential risk factor)	Criteria for HTN Diagnosis	Data collection tool and methods
Getahun et al. (2010) [57]	Ethiopia	648 adults, 35–65 age group and random selection	Cross sectional	Khat (Catha edulis) chewing	≥ 140/90 mmHg	A semi-structured questionnaire and Blood pressure measurement
Mossie et al. (2002) [58]	Ethiopia	898 adults, 16 years and above and random selection	Cross sectional	Khat (Catha edulis) chewing	≥ 140/90 mmHg	A semi-structured questionnaire and Blood pressure measurement
Ibrahim et al. (2011) [80]	Saudi Arabia	1775 adults aged 16 years and above and Random selection	Cross sectional	Khat (Catha edulis) chewing	≥ 140/90 mmHg	A semi-structured questionnaire and Blood pressure measurement
Ayehu et al. (2020) [84]	Ethiopia	222 adults aged 20 years and above and Random selection	Cross sectional	Khat (Catha edulis) chewing	≥ 140/90 mmHg	A structured questionnaire and Blood pressure measurement
Chukaeta et al. (2020) [73]	Ethiopia	3346 adults aged 25 years and above and Random selection	Cross sectional	Khat (Catha edulis) chewing	≥ 140/90 mmHg	A structured questionnaire and Blood pressure measurement
Geta et al. (2019) [74]	Ethiopia	1037 adults aged 18 years and above and Random selection	Cross sectional	Khat (Catha edulis) chewing	≥ 140/90 mm/Hg	A structured questionnaire and Blood pressure measurement
Gebremedhin et al. (2021) [83]	Ethiopia	136 cases and 270 controls adults aged 18 and above and consecutive sampling.	Unmatched case control	Khat (Catha edulis) chewing	≥ 140/90 mmHg	A structured questionnaire and Blood pressure measurement
Badego et al. (2020) [75]	Ethiopia	546 adults aged 18 years and above and Random selection	Cross sectional	Khat (Catha edulis) chewing	≥ 140/90 mmHg	A structured questionnaire and history of hypertension or taking antihypertensive drug
Yimam et al. (2021) [76]	Ethiopia	275 adults aged 21 years and above and Random selection	Cross sectional	Khat (Catha edulis) chewing	≥ 140/90 mmHg	A structured questionnaire and Blood pressure measurement
Abdissa and Kene et al. (2019) [77]	Ethiopia	366 adults aged 18 years and above and Random selection	Cross sectional	Khat (Catha edulis) chewing	≥ 140/90 mmHg	A structured questionnaire and Blood pressure measurement
Wachamo et al. (2020) [78]	Ethiopia	383 adults aged 18 years and above and Random selection	Cross sectional	Khat (Catha edulis) chewing	≥ 140/90 mmHg	A structured questionnaire and Blood pressure measurement
Sisay et al. (2014) [68]	Ethiopia	701 adults aged 18 years and above and Random selection	Cross sectional	Khat (Catha edulis) chewing	≥ 140/90 mmHg	A semi-structured questionnaire and Blood pressure measurement
Seifu W, et al. (2017) [69]	Ethiopia	330 adults aged 20 years and above and Random selection	Cross sectional	Khat (Catha edulis) chewing	≥ 140/90 mmHg	A structured questionnaire and history of hypertension or taking antihypertensive drug
Motuma et al. (2023) [79]	Ethiopia	1164 adults aged 18 years and above and Random selection	Cross sectional	Khat (Catha edulis) chewing	≥ 140/90 mmHg	A structured questionnaire and Blood pressure measurement
Hassen and Mamo et al. (2019) [71]	Ethiopia	312 adults aged 18 years and above and Random selection	Cross sectional	Khat (Catha edulis) chewing	≥ 140/90 mmHg	A structured questionnaire and Blood pressure measurement

### Association between Khat (Catha edulis) chewing and hypertension

From overall meta-analysis, a total of 12,409 participants were involved. Of the 3986 Khat chewers, 1278 were found to have hypertension. On contrary, out of the 8423 of non-chewers, 1341 were found to have hypertension. The pooled association between hypertension and Khat chewing was estimated using 15 studies. The OR was used to estimate the association. The OR ranges from 0.23 (95% CI; 0.11–0.47) to 17.39(95%CI; 6.17–49.04). The result showed a high level of heterogeneity (Heterogeneity test: Tau² = 0.81; Chi² = 205.33, df = 14 (*P* < 0.00001); I² = 93% (95% CI: 89–96%)).

**Fig. 2:**
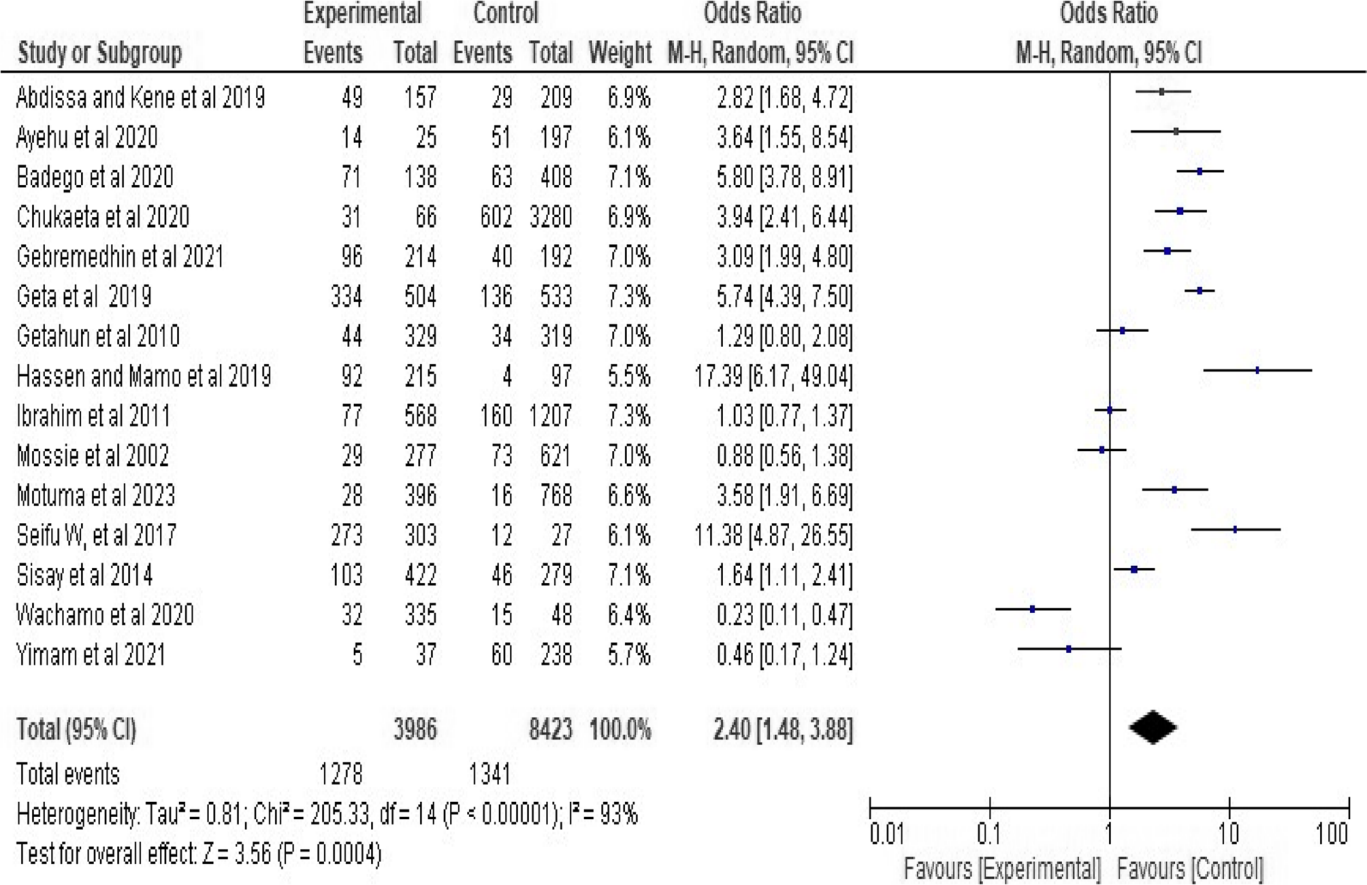
Forest plot of the included studies showing the association between Khat chewing and hypertension from random-effect model analysis, 2024

Due to this, the pooled association between hypertension and khat chewing was determined using a random-effects model. Therefore, increased likelihood of hypertension was identified among individuals who chew khat. Accordingly, individual who chew khat were 2.4 times more likely to have hypertension as compared to individuals who never chew khat (OR 2.4, 95%CI; 1.48–3.88) at *p* = 0.0004. (See Fig. 2). Regarding studies weight, the highest weight among studies observed from the studies conducted by Geta et al. [74] and Ibrahim et al. [80].

### Publication bias

**Fig. 3:**
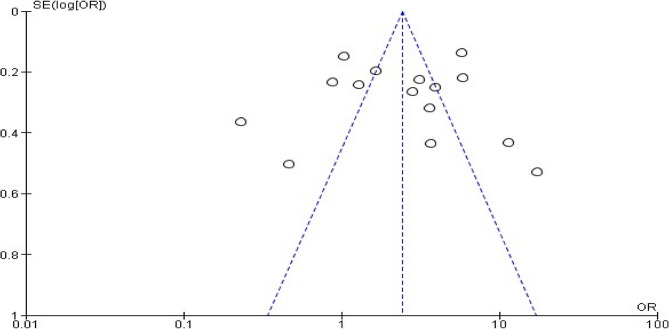
Funnel plot on the association between hypertension and Khat chewing, 2024

The funnel plot revealed a symmetrical distribution (Fig. 3). The Egger’s linear regression test was used to objectively identify publication bias. Egger’s linear regression test was not statistically significant (*P* = 0.655) (Table 4). Therefore, Egger’s linear regression test revealed no evidence of publication bias.

**Table 4:** Egger’s test for small-study effects for the studies of hypertension and Khat chewing, 2024

Number of studies = 15 Root MSE = 2.664					
Standard effect	Coefficient	Standard error	t	P-value	95%CI
Slope	0.7576267	0.4046606	1.87	0.084	− 0.11658993,1.631843
Bias	− 0.830552	1.816923	−0.46	***0.655***	−4.755775,3.094671

### Heterogeneity of the studies

Significant heterogeneity observed from the pooled random effects model estimate. To handle this heterogeneity, sensitivity analysis and subgroup analysis were performed.

### Sensitivity analysis

**Fig. 4:**
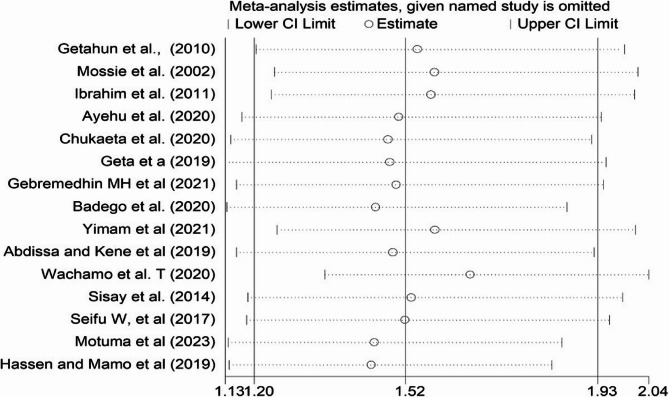
Sensitivity analysis plot for the pooled association between hypertension and khat chewing, 2024

Sensitivity analysis was performed to evaluate the effect of individual studies on the pooled random effect estimate. When individual study was omitted, the pooled effect size obtained was within the 95% CI of the overall pooled random effect estimate. This confirms the absence of single study impact on the overall pooled random effect estimate. Therefore, from the random effect model, there are no studies that excessively influence the overall pooled estimate of association between hypertension and Khat (Fig. 4).

### Sub-group analysis

Subgroup analysis conducted by year of publication.

### Pooled association by year of publication

**Fig. 5:**
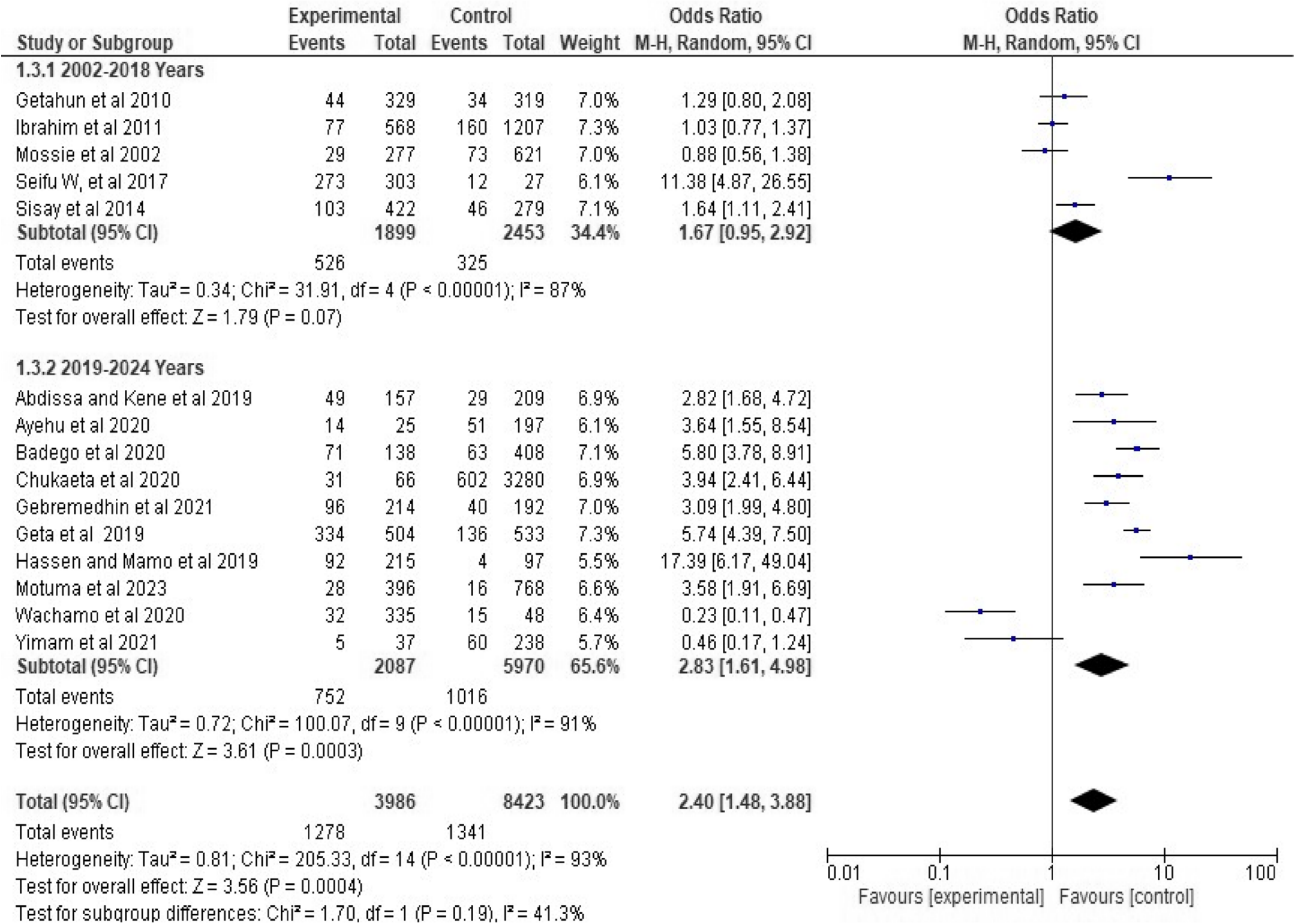
Forest plot showing the subgroup analysis for pooled association between hypertension and khat chewing, 2024

Among 15 studies in the random effects model, five studies conducted between 2002 and 2018 years showed that there is no pooled association between hypertension and khat chewing study (OR 1.67, 95%CI; 0.95–2.92) at *p* = 0.07. Additionally, ten studies conducted between 2019 and 2023 years pooled and showed that statistically significant association between hypertension and khat chewing. Accordingly, khat chewer were 2.83 times more likely to have hypertension as compared to individuals who never chew khat (OR 2.83, 95%CI; 1.61–4.98) at *p* = 0.0003. Furthermore, both groups have high heterogeneity and study conducted between 2002 and 2018 years showed heterogeneity test (Heterogeneity: Tau² = 0.34; Chi² = 31.91, df = 4 (*P* < 0.00001); I² = 87% (95% CI: 70–95%)) and studies conducted between 2019 and 2023 years showed heterogeneity test (Heterogeneity: Tau² = 0.81; Chi² = 205.33, df = 14 (*P* < 0.00001); I² = 93% (95% CI: 88–96%)) respectively (See Fig. 5).

#### Certainty of evidence using GRADE approach

The overall certainty of evidence for association between Khat chewing and hypertension is rated as low (Table 5). This suggests that future studies should employ prospective cohort designs with rigorous control for confounders (e.g., matched controls, adjustment for diet, physical activity, and genetic factors) and minimize recall bias through objective khat-use assessments (e.g., biochemical validation). Researchers should standardize exposure definitions (e.g., dose, frequency, duration of khat use) and additional outcome measurements methods (e.g., ambulatory blood pressure monitoring instead of only single readings). Future research should prioritize multicenter studies involving diverse populations to distinguish whether observed heterogeneity arises from methodological inconsistencies or genuine regional variations in khat’s physiological effects. Employing standardized diagnostic tools across high-quality, geographically representative studies would reduce variability and enhance the precision of effect estimates. As per GRADE guidelines, such rigorous investigations are likely to strengthen confidence in the findings and could significantly refine current conclusions.

**Table 5:** Certainty assessment using GRADE approach for pooled association between hypertension and khat chewing, 2024

Outcome	Domain	Assessment	Certainty of evidence
Hypertension incidence	Risk of bias	All included studies were observational (cross-sectional or case-control), which inherently carry a higher risk of bias compared to randomized controlled trials (RCTs). Factors such as recall bias, selection bias, and confounding variables (e.g., age, lifestyle factors, genetic predisposition) were not fully controlled for in these studies.	Downgrade by 1 level for serious risk of bias
	Inconsistency	High statistical heterogeneity was observed (I² = 93%), indicating substantial variability in effect sizes across studies. Subgroup analysis by publication year reduced but did not eliminate this inconsistency	Downgrade by 1 level for serious inconsistency
	Indirectness	The studies directly addressed the research question, and hypertension was measured using standardized diagnostic criteria (BP ≥ 140/90 mmHg). No surrogate outcomes were used.	No downgrade (evidence is direct)
	Imprecision	The pooled estimate had a narrow confidence interval (OR 2.4, 95% CI 1.48–3.88), suggesting certainty in the effect size. Additionally, the large sample size (12,409 participants) provided sufficient statistical power.	No downgrade (imprecision is not severe enough to warrant lowering certainty).
	Publication bias	Funnel plot symmetry and Egger’s test (*P* = 0.655) indicated no significant publication bias	No downgrade (no evidence of small-study
	Other GRADE Considerations	The odds ratio of 2.4 indicates a moderate-to-strong association between khat chewing and hypertension. Additionally, a dose-response relationship was observed, with longer chewing sessions correlating with higher hypertension risk, supporting biological plausibility. This aligns with the known pharmacological effects of cathinone, khat’s active compound, which increases sympathetic nervous system activity and elevates blood pressure. These factors strengthen confidence in the observed association despite limitations in study designs.	Upgrade by 1 level due to large effect size and dose-response relationship.
Final certainty			Low certainty

## Discussion

This systematic review and meta-analysis aimed to explore the evidence on the epidemiologic association of Khat chewing as risk factor on hypertension. The study synthesizes data from 15 studies involving 12,409 participants, providing a substantially broader evidence base compared to earlier reviews [85]. The current study concludes that khat chewers are 2.4 times more likely to develop hypertension than non-chewers (OR 2.4, 95% CI; 1.48–3.88), with a noted dose-response relationship. This study addressed limitations of earlier reviews by including a larger number of studies and employing rigorous methods such as subgroup and sensitivity analyses to manage heterogeneity.

In contrast, the 2012 review by Hassen et al. [85] analyzed only three studies (3,321 participants) and found no significant association (OR 1.04, 95% CI 0.84–1.29), attributing this to insufficient data and methodological limitations. Additionally, the review conducted by Mega and Dabe (2017) [37] analyzed 10 studies (9,207 participants) and reported that khat chewers had significantly higher systolic and diastolic blood pressure and heart rates compared to non-chewers, but their review was limited by high heterogeneity and the inclusion of fewer studies.

Compared to previous 2012 and 2017 review [37, 85], the current review employs advanced statistical techniques, including random-effects models and GRADE assessments, and addresses heterogeneity through subgroup analyses. It also incorporates pharmacological evidence to explain the role of cathinone in sustained blood pressure elevation. Moreover, the current review includes studies up to 2023, capturing more recent and geographically diverse research. This broader temporal scope strengthens its conclusions. Conversely, the 2012 and 2017 review [85] was limited to studies up to 2011 and 2017 respectively, missing later research that could have reinforced the association between khat chewing and hypertension. Policymakers should consider khat use as a modifiable risk factor in hypertension prevention programs. Future research should conduct randomized controlled trials (RCTs) or cohort studies to establish causality and use objective biomarkers (e.g., blood cathinone levels) to reduce recall bias.

Overall, the findings of this study suggests that there is evidence of association between Khat chewing and hypertension. The finding showed that individual khat chewers were 2.4 times more likely to have hypertension as compared to individuals with non-khat chewers. This finding is supported by a previous study, which reported that Khat chewers have were 1.42 more likelihood to develop hypertension than non-khat chewers [86]. It is also supported by another study, which reported that khat chewing as risk factors for hypertension and other cardiovascular disease [87].

This might be due to Khat contains chemicals cathinone, cathine, and amphetamine. Cathinone is structurally related to amphetamine which increases levels of dopamine in the brain by acting on the catecholaminergic synapses and releases of noradrenaline, which might have a sustained effect as a peripheral vasoconstrictor among khat chewers [22, 30, 88] and as a result increase of blood pressure and heart rate [17, 22, 29]. The 2.4-fold increased odds of hypertension among khat chewers highlight an urgent need for policy interventions, such as regulating khat sales and integrating khat cessation into hypertension management programs. The future research should investigate dose-response relationships (e.g., chewing duration, frequency) to refine risk stratification.

Subgroup analysis between 2019 and 2024 years also suggests that there is evidence of association between Khat chewing and hypertension. The finding showed that individual who chew khat were 2.83 times more likely to have hypertension as compared to individuals with non-khat chewers. However, subgroup meta-analysis between 2002 and 2018 years suggest that there is no association between khat chewing and hypertension. The contrast might be due number of studies, and sample size included in the study. Both groups in subgroup analysis revealed high heterogeneity.

This systematic review and meta-analysis have some strengths and limitations. Our review incorporates thirteen additional studies examining the association between Khat chewing and hypertension, providing a more up-to-date synthesis than the previous review conducted in 2012 [36]. It also included frequency of khat chewing data which did not included in the first review. All included studies use the same definition to declare hypertension. Subgroup analysis was performed to minimize statistical heterogeneity.

However, this systematic review and meta-analysis on khat chewing and hypertension primarily included cross-sectional studies, which are prone to potential confounding, selection bias, recall bias, social desirability bias, potentially skewing self-reported data and the inability to establish causality. The absence of randomized controlled trials (RCTs) or quasi-experimental designs means that observational studies which inherently lack random assignment cannot establish causality with high certainty. Without randomization, confounding variables (e.g., lifestyle habits, genetic predispositions) remain uncontrolled, weakening the reliability of causal conclusions.

The current study also focuses on hypertension but lacks heart rate data, which is a key cardiovascular effect of khat due to cathinone-induced sympathetic activation. Although this study confirms a strong association between khat use and hypertension, the absence of heart rate measurements limits a complete assessment of khat’s cardiovascular impact.

Heterogeneity across studies (I² = 93%) may stemming from variations in khat dosage, duration of use, and diagnostic criteria for hypertension further undermines the reliability of pooled estimates. Additionally, only full text articles published in the English language were included in the review and therefore key articles possibly published in other languages especially in Arabic may have been omitted as Khat is highly consumed in the east coast of Africa and the Arabian Peninsula where Arabic is the common language. Furthermore, Scopus, CINAHL, EMBASE and Web of Sciences databases were excluded because access to them was restricted in Ethiopia.

### Conclusion and recommendations

The findings of this systematic review and meta-analysis underscore the significant public health implications of khat chewing as a modifiable risk factor for hypertension, particularly in regions where its use is prevalent. The robust association between khat chewing and hypertension, supported by pharmacological evidence of cathinone’s vasoconstrictive effects, calls for targeted interventions, including public health campaigns to raise awareness about cardiovascular risks and policy measures to regulate khat sales. Given the dose-response relationship, healthcare systems should integrate khat cessation counseling into hypertension prevention and management programs, especially in high-prevalence areas. Addressing khat use as a preventable contributor to hypertension could reduce the burden of cardiovascular disease and improve population health outcomes.

To strengthen the evidence on the association between khat chewing and hypertension, future studies should prioritize rigorous experimental designs, such as randomized controlled trials (RCTs) or longitudinal cohort studies, to establish causality and minimize confounding bias. Standardized protocols for measuring khat consumption (e.g., dosage, frequency, duration) and hypertension diagnosis are essential to reduce heterogeneity and improve comparability across studies.

Additionally, expanding literature searches to include non-English publications (particularly Arabic) and accessing major databases (e.g., Scopus, CINAHL and EMBASE) would enhance the comprehensiveness of evidence, given khat’s prevalence in Arabic-speaking regions. Researchers should also employ objective biomarkers (e.g., blood cathinone levels) alongside self-reports to mitigate recall and social desirability biases. Collaborative efforts with institutions in khat-endemic regions could facilitate access to restricted databases and underrepresented populations, ensuring more generalizable findings. Moreover, future studies should incorporate heart rate monitoring alongside blood pressure measurements to fully evaluate khat’s cardiovascular effects, given its known stimulant properties.

*Protocol Registration*: This systematic review and meta-analysis was registered in PROSPERO with the registration ID and link as follows: CRD42024555322: Available from: https://www.crd.york.ac.uk/prospero/display_record.php.

## Supplementary Information

Supplementary Material 1

## Data Availability

No datasets were generated or analysed during the current study.

## Abbreviations

AJOL: African Journals Online
AOR: Adjusted Odd Ratio
BMI: Body Mass Index
CI: Confidence Interval
CVDs: Cardiovascular Diseases
DALYs: Disability-Adjusted Life-Years
HTN: Hypertension
JBI: Joanna Briggs Institute
MeSH: Medical Subject Headings
PRISMA-P: Preferred Reporting Items for Systematic Review and Meta-Analysis Protocols
